# Cold-inducible RNA-binding Protein Induces Neutrophil Extracellular Traps in the Lungs during Sepsis

**DOI:** 10.1038/s41598-019-42762-1

**Published:** 2019-04-18

**Authors:** Yasumasa Ode, Monowar Aziz, Hui Jin, Adnan Arif, Jonathan G. Nicastro, Ping Wang

**Affiliations:** 1 Center for Immunology and Inflammation, The Feinstein Institute for Medical Research, Manhasset, NY USA; 2 Department of Surgery and Molecular Medicine, Donald and Barbara Zucker School of Medicine at Hofstra/Northwell, Manhasset, NY USA

**Keywords:** Inflammation, Acute inflammation

## Abstract

Extracellular cold-inducible RNA-binding protein (CIRP) exaggerates inflammation and tissue injury in sepsis. Neutrophil extracellular traps (NETs) are released by activated neutrophils during sepsis. NETs contribute to pathogen clearance, but excessive NET formation (NETosis) causes inflammation and tissue damage. Peptidylarginine deiminase 4 (PAD4) is associated with NETosis by increasing histone citrullination and chromatin decondensation. We hypothesized that CIRP induces NETosis in the lungs during sepsis via upregulating PAD4 expression. Sepsis was induced in C57BL/6 wild-type (WT) and CIRP^−/−^ mice by cecal ligation and puncture (CLP). After 20 h of CLP induction, NETs in the lungs of WT and CIRP^−/−^ mice were quantified by flow cytometry by staining the single cell suspensions with MPO and CitH3 Abs. PAD4 expression in the lungs of WT and CIRP^−/−^ mice after sepsis was assessed by Western blotting. *In vitro* effects of recombinant mouse (rm) CIRP for NETosis and PAD4 expression in the bone marrow-derived neutrophils (BMDN) were assessed by flow cytometry and Western blotting, respectively. After 20 h of CLP, NETosis in the lungs was significantly decreased in CIRP^−/−^ mice compared to WT mice, which also correlated with the decreased PAD4 expression. Intratracheal administration of rmCIRP into WT mice significantly increased NETosis and PAD4 expression in the lungs compared to vehicle-injected mice. *In vitro* culture of BMDN with rmCIRP significantly increased NETosis and PAD4 expression compared to PBS-treated control. Fluorescence microscopy revealed typical web-like structures consistent with NETs in rmCIRP-treated BMDN. Thus, CIRP serves as a novel inducer of NETosis via PAD4 during sepsis.

## Introduction

Sepsis is defined as a life-threatening organ dysfunction caused by a dysregulated host response to infection^1^. The lungs are particularly susceptible to injury in sepsis, and approximately 50% of patients with sepsis develop acute lung injury (ALI) or acute respiratory distress syndrome (ARDS)^2^. Sepsis morbidity and mortality are dramatically elevated by ALI^2^. Except antibiotics and infectious source control, management of the patients with sepsis-induced ALI is largely restricted to supportive therapies^3^, partly due to an incomplete understanding of their pathophysiology.

In inflammation, cold-inducible RNA-binding protein (CIRP) is released into the extracellular space^4^. Extracellular CIRP is a damage-associated molecular pattern (DAMP) in sepsis and leading to organ injury and increased mortality rates^4^. Elevated plasma levels of CIRP were later independently correlated with poor prognosis among patients with sepsis^5^. CIRP is a constitutively-expressed nuclear chaperone involved in protein translation^6^. When cells are exposed to stressors such as lipopolysaccharide (LPS), hypothermia, hypoxia, and UV irradiation, CIRP is upregulated, translocated from the nucleus to cytoplasmic stress granules, and released to the extracellular space^4,6,7^. Once outside the cell, CIRP promotes proinflammatory responses via its binding to the Toll-like receptor (TLR4) and myeloid differentiation factor 2 (MD2) complex on macrophages^4^. We have recently shown that CIRP is increased in the lungs during sepsis^8^. We also found that CIRP-neutralizing antibodies (Ab) ameliorate sepsis^4^, CIRP^−/−^ mice are protected from sepsis and ALI^9^, and healthy mice injected with recombinant mouse (rm) CIRP develop lung injury^10^. Together, these findings indicate that CIRP is a major player in the pathogenesis of sepsis and ALI. However, how CIRP increases inflammation and injury during sepsis and ALI remains to be fully explained.

One of the hallmark features of ALI is increased influx of neutrophils into the lungs. A recent advance in leukocyte biology was the discovery of neutrophil extracellular traps (NETs)^11^. NETs consist of chromatin filaments coated with citrullinated histone H3 (CitH3) and neutrophil enzymes such as myeloperoxidase (MPO) and elastase, which are released by neutrophils upon stimulation with LPS, phorbol 12-myristate 13-acetate (PMA), N-formylmethionyl-leucyl-phenylalanine (fMLP), or interleukin-8 (IL-8)^12,13^. Although NETs contribute to pathogen clearance, excessive NET formation in sepsis has a deleterious effect by promoting tissue damage and thereby perpetuating inflammation^12,14,15^. CIRP and NETs are both elevated in sepsis^4,15,16^. However, NET formation has not yet been studied in relation with CIRP during sepsis.

Peptidylarginine deiminases 4 (PAD4) is expressed in granulocytes and catalyzes citrullination of histones, leading to chromatin decondensation which is essential for NETosis^13^. PAD4 not only induces chromatin decondensation, but also helps release chromosomal DNA into the extracellular space^17,18^. PAD4 deficient mice were shown to be protected from lung injury in hemorrhagic shock and sepsis^16^. Similarly, targeting PAD4 by its inhibitor significantly decreased NET formation, and improved survival in murine sepsis^15^. Although the impact of PAD4 in NETosis in sepsis and ALI has been implicated, CIRP’s role in inducing PAD4 expression and NETosis in the lungs during sepsis has not been determined yet.

Therefore in the present study, we aimed to study the effect of CIRP on NETosis in the lungs during sepsis. In order to do this, we first induced sepsis in WT and CIRP^−/−^ mice and then compared the status of NETosis and PAD4 expression in their lungs. To determine the direct effect of CIRP, we administered rmCIRP into the mice and then assessed NETosis and PAD4 expression in the lungs. To support these *in vivo* findings, we also stimulated murine primary neutrophils with rmCIRP and then assessed NETosis and PAD4 expression. Our data showed that CIRP could induce NET formation and PAD4 expression in the lungs in mice during sepsis, thereby revealing a new drug candidate by targeting CIRP to control sepsis and ALI through the downregulation of PAD4-dependent NETosis in the lungs during sepsis.

## Materials and Methods

### Mice

Male 8-12 week old wild-type (WT) C57BL/6 mice were purchased from Charles River (Charles River, Wilmington, MA). C57BL/6 CIRP^−/−^ mice were obtained from Jun Fujita (Kyoto University, Kyoto, Japan). Mice were housed in rooms at an ambient (~23 °C) temperature with 12 h light cycles and provided standard laboratory chow and drinking water. All experiments were performed in accordance with the guidelines for the use of experimental animals by National Institutes of Health (Bethesda, MD). All the experiments involving mice were approved by the Institutional Animal Care and Use Committees (IACUC) of the Feinstein Institute for Medical Research.

### Mouse model of polymicrobial sepsis

WT and CIRP^−/−^ mice were anesthetized with 2% isoflurane inhalation. The abdomen was shaved and made sterile with 10% povidone-iodine wash. Sepsis was induced in mice by cecal ligation and puncture (CLP)^19^. Briefly, after a 1.5 cm midline abdominal incision the cecum was exposed and ligated at 1 cm from the tip with 4-0 silk suture. Ligated distal part of the cecum was punctured once through and through using a 22-g needle and squeezed gently to extrude a small amount of fecal material. The cecum was positioned back inside the abdomen, and the abdominal wound was closed in two layers with running 6-0 silk suture. Sham mice underwent only abdominal incision without ligation and puncture of the cecum. Both sham- and CLP-operated animals were resuscitated with 1 ml of normal saline subcutaneously to avoid their dehydration caused by surgical stress, and then returned to their cages with normal access to foods and drinking water for 20 h.

### Isolation of cells from lung tissues

Before extraction, lungs were perfused with 30 ml normal saline through right ventricle of the heart under 2% isoflurane inhalation anesthesia. Lung tissues were harvested from mice and filled with Ca^++^ and Mg^++^ free Hank’s balanced salt solution (HBSS) (Corning; Corning, NY).

They were sliced into small pieces by sterile surgical blade and were digested in HBSS supplemented with 100 U/ml of collagenase I (Worthington Biochemical, Lakewood, NJ) for 30 min at 37 °C with periodic shaking. The resultant fragments were meshed by a 70 µm cell strainer (Corning) and a 10 ml syringe plunger and washed with HBSS. The remaining red blood cells were lysed with lysing buffer (BD Biosciences, San Jose, CA). The numbers of isolated lung cells were counted using a microscope (Eclipse TS100; Nikon, Tokyo, Japan).

### Quantification of neutrophil extracellular traps (NETs) by flow cytometry

NETs were detected by flow cytometry as described previously with minor modifications^20^.

In brief, single-cell suspensions were fixed with 2% paraformaldehyde for 20 min at room temperature, blocked for 30 min with 2% bovine serum albumin (BSA) in PBS at 37 °C. Cells were surface-stained with APC-rat anti-mouse Ly-6G Ab (Clone: 1A8; Biolegend, San Diego, CA), FITC-mouse anti-myeloperoxidase (MPO) Ab (Clone: 2D4; Abcam, Cambridge, MA) and rabbit anti-histone H3 (CitH3) Ab (citrulline R2 + R8 + R17; Cat. No ab5103: Abcam) followed by staining with PE-donkey anti-rabbit IgG (Clone: Poly4064; Biolegend). To avoid staining of intracellular chromatin and enzymes, cells were not permeabilized. More than 10,000 events were acquired using a BD LSR Fortessa Flow Cytometry Analyzer (BD Biosciences) and the data were analyzed by FlowJo software (Tree Star, Ashland, OR). In the flow cytometry dot plots, at first the cells were gated based on their size and intracellular granularity by forward and side scatters, respectively. Next, neutrophils were gated based on Ly6G positive staining. In the Ly6G positive population, we then gated the cells based on surface/extracellular MPO and citH3 staining. We chose quadra/rectangular gate to select MPO^+^citH3^+^ in Ly6G^+^ neutrophils to define the NETs^+^ neutrophils. Purified rabbit polyclonal IgG (clone: Poly29108, Biolegend) and FITC-mouse IgG1 (B11/5, ab91356, Abcam) were used as the isotype controls.

### Recombinant murine CIRP injection in mice

Recombinant murine CIRP (rmCIRP) was prepared in-house and the quality control assays were performed as described previously^4^. The quality of the purified protein was assessed by Western blotting and the functional assay of the protein was done by assessing the TNFα levels in the macrophages. The level of LPS in the purified protein was measured by a limulus amebocyte lysate (LAL) assay (Cambrex, East Rutherford, New Jersey). Only the purified protein lots that were endotoxin free were considered for *in vitro* and *in vivo* experiments. We performed these quality control assays for each purified protein lot. To rule out a contribution from LPS in the inflammatory response to rmCIRP, our previous study showed that incubation with polymyxin B, an LPS-binding antibiotic, did not interfere with rmCIRP-induced production of TNF-α, whereas heat treatment reduced the activity of rmCIRP in macrophages^4^. WT mice were anesthetized with 2% isoflurane inhalation, and 3 mg/kg BW of rmCIRP or the same amount of vehicle (normal saline) was carefully injected intratracheally (*i.t*.). After 5 h, under 2% isoflurane inhalation anesthesia, lungs were perfused with 30 ml of normal saline through the right ventricle of the heart and harvested for the various analyses.

### Isolation and purification of bone marrow-derived neutrophils (BMDN)

The protocol for the isolation and purification of BMDN was followed from our previous study^8^. Mice were anesthetized by 2% isoflurane inhalation and the femurs and tibias were dissected. Bone marrow contents were flushed out with Ca^++^ and Mg^++^ free HBSS into a petri dish using a 25 gauge needle. Cell suspensions were filtered through a 70 µm cell strainer (Corning) and BMDN were purified by negative selection using the EasySep mouse neutrophil enrichment kit (Cat No.: 19762 and 18000; STEMCELL, Vancouver, Canada). The purity of sorted neutrophils was checked by staining the cells with Ly6G (clone 1A8, Biolegend) and CD11b (clone M1/70, Biolegend) Abs using BD LSR Fortessa flow cytometer (BD Biosciences). After purification, we obtained 99% of the purity of the cells (Supplemental Fig. 1).

### *In vitro* stimulation of BMDN with recombinant mouse CIRP

A total of 1 × 10^6^ (for flow cytometry) or 2 × 10^6^ (for Western blotting) BMDN were cultured in RPMI 1640 medium (ThermoFisher Scientific, Waltham, MA) and stimulated with various doses of rmCIRP for 4 h at 37 °C in 5% CO_2_ humidified incubator, and then subjected to NETs assay by flow cytometry and assessed for PAD4 expression by Western blotting.

### NETs assay by fluorescence microscope

A total of 1 × 10^5^ BMDN grown in culture slides (Cat. No.: 177402; ThermoFisher Scientific, Waltham, MA) were treated with rmCIRP (1 µg/ml) for 4 h at 37 °C in 5% CO_2_. The cells were fixed with 4% paraformaldehyde and stained with 2 µM SYTOX green (Cat No.: S7020; ThermoFisher scientific) for extracellular DNA detection, followed by the assessment using fluorescence microscope (Eclipse Ti-S; Nikon).

### Assessment of PAD4 expression in lungs or BMDN by Western blotting

Harvested lungs or rmCIRP-treated BMDN were homogenized in lysis buffer containing 10 mM Tris pH 7.5, 1% TritonX-100, 1 mM EDTA, 1 mM EGTA, 2 mM Na_3_VO_4_, 0.2 mM PMSF, 2 µg/ml aprotinin and protease inhibitor cocktail (Roche Diagnostics, Indianapolis, IN) by sonication (Sonic Dismembranator100; Fisher Scientific, Pittsburgh, PA). Concentration of protein of each sample was determined by Bio-Rad protein assay reagent (Bio-Rad, Hercules, CA). Equal amounts of protein from lung homogenates or cell lysates were fractionated on Bis-Tris gels (4–12%) and transferred to a 0.2 µm pore size nitrocellulose membrane. The membrane was blocked with 0.1% casein in Tris-buffered saline with 0.1% tween-20 (TBST) and incubated with anti-PAD4 Ab (Cat No: 17373-1-AP; Proteintech, Rosemont, IL) or β-actin primary Ab (Cat No: A5441; Sigma-Aldrich, St Louis, MO). After washing the membranes with TBST buffer, they were incubated with fluorescent-labeled secondary Abs (Li-Cor Biosciences, Lincoln, NE). Bands were detected by Odyssey FC Dual-Mode Imaging system (Li-Cor Biosciences) and the intensities of bands were measured using Image J software. The SeeBlue® Plus2 Pre-Stained Standard (LC5925, ThermoFisher Scientific) protein molecular weight marker was used to determine the target protein and evaluate Western blot transfer efficiency.

### PAD4 activity assay

The *in vitro* PAD4 activity assay was performed colorimetrically as described previously^21^. BMDN (1 × 10^6^ cells) were lysed in lysis solution [50 mM Tris, 50 mM NaCl, 0.1 mM phenylmethylsulfonyl fluoride (PMSF, MW 174.94 g/mol) and 1 mM dithiothreitol (DTT, MW 154.25 g/mol), pH = 8.0] by sonication. Equal amount of protein lysates in 60 μl volume was taken in a black-walled, clear-bottom 96-well microplate (ThermoFisher Scientific) and then added 100 μl of solution A (100 mM NaCl, 100 mM Tris, 20 mM CaCl_2_, and 2 mM TCEP, pH = 8.0) into all wells and incubated for 20 min at RT. We next added 40 μl of solution B [ZRcoum (Z-Arg-Arg-7-amido-4-methyl-coumarin hydrochloride) (250 μM), Sigma Aldrich, St. Louis, MO, USA] into all wells and incubated for 45 min at RT. Finally, we added 10 μl of solution C (10 mg/ml trypsin in 100 mM EDTA) to all wells and incubated at RT for 10 min. The fluorescence was recorded on a multimodal reader (BioTek, Synergy H1 Hybrid Reader, Winooski, VT, USA) with an excitation wavelength of 345 nm and an emission wavelength of 465 nm. The PAD4 activity was expressed as units/mg of protein, in which 1 unit was defined as 1/change of fluorescence (with trypsin − without trypsin)/min.

### Statistical analysis

Data are presented as mean ± standard error of the mean (SEM). Differences between two groups were assessed with the unpaired Student’s *t* test. The p values < 0.05 were considered statistically significant. Multiple groups were compared by one-way analysis of variance (ANOVA) using the Student-Newman-Keuls test. All statistical tests were performed and graphics were created with Sigma Plot graphing and statistical analysis software (Systat Software Inc., San Jose, CA).

## Results

### Decreased NETosis in the lungs of CIRP-deficient mice during sepsis

**Figure 1:**
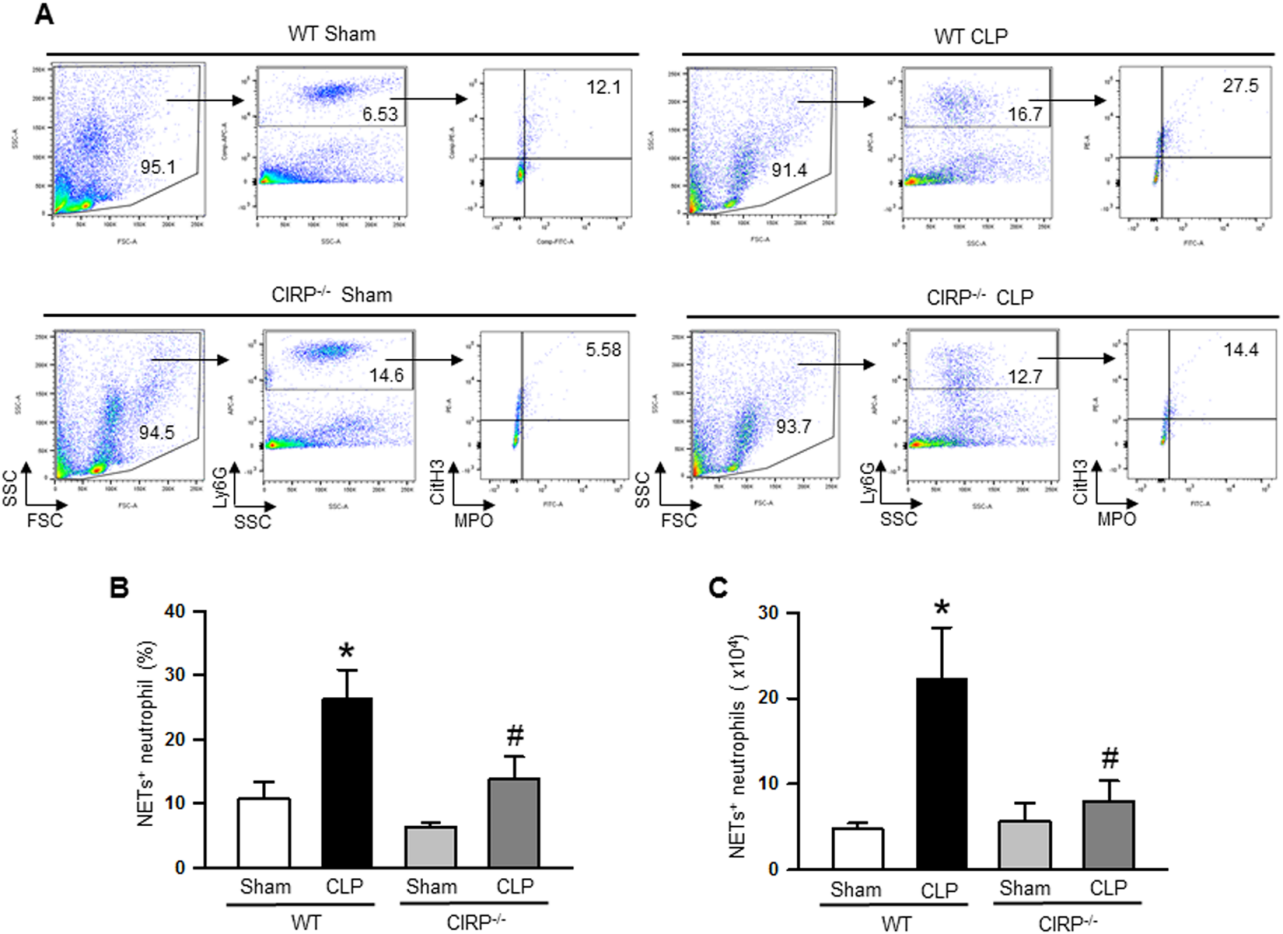
Assessment of NETs in the lungs during sepsis. At 20 h of CLP or sham operation, lungs were perfused and harvested from WT or CIRP^−/−^ mice. NET forming neutrophils in the lungs was detected by flow cytometry by staining the single cell suspension with anti-mouse Ly6G, MPO, and CitH3 Abs. (**A**) Representative dot plots of the frequencies of NET forming neutrophils in lungs from WT and CIRP^−/−^ mice generated from three independent experiments are shown. Bar diagrams representing the quantitative mean values of the **(B)** frequencies and **(C)** numbers of NET forming neutrophils in the lungs are shown. Data are expressed as means ± SE (n = WT sham 4, WT CLP 6, CIRP^−/−^ sham 6, and CIRP^−/−^ CLP 6 mice) and compared using one-way ANOVA and SNK method (*p < 0.05 vs WT sham, ^#^p < 0.05 vs WT CLP).

Based on our previous studies^22,23^, which represented the dynamics of the hyperinflammation phase, we chose the 20 h time point after CLP to assess NETs and PAD4 levels in the lungs. Our previous studies showed dramatic increase in the expression of proinflammatory cytokines, chemokines and infiltration of neutrophils at 20 h of CLP, causing severe systemic inflammation and injury and inflammation at the lungs^22,23^. Other than the 20 h time point after CLP, we also assessed NETs in the lungs of WT mice at 10 h after CLP. We found that at 10 h after CLP the frequencies of NET-forming neutrophils were increased as compared to sham mice. However, the highest increase in the frequencies of NET-forming neutrophils occurred at 20 h after CLP as compared to sham as well as 10 h after CLP (Supplemental Fig. 2). Therefore, to determine the link between CIRP and NETs we performed CLP in WT and CIRP^−/−^ mice and after 20 h of CLP, we assessed NETs and PAD4 expression in the lungs. After 20 h of CLP induction, the frequencies of NET-forming neutrophils in the lungs of WT mice were significantly increased by 2.4 fold as compared to the sham-operated mice **(**Fig. 1A,B). In contrast, the frequencies of NET-forming neutrophils in the lungs of CIRP^−/−^ mice were significantly decreased by 48% compared to the WT septic mice **(**Fig. 1A,B). Similarly, we also found that the total numbers of NET-forming neutrophils in the lungs were increased in the WT mice following sepsis, while the numbers of NET-forming neutrophils in the lungs of CIRP^−/−^ mice were significantly decreased by 64% as compared to WT septic mice **(**Fig. 1C).

### *In vivo* administration of rmCIRP induces NETosis in the lungs

**Figure 2:**
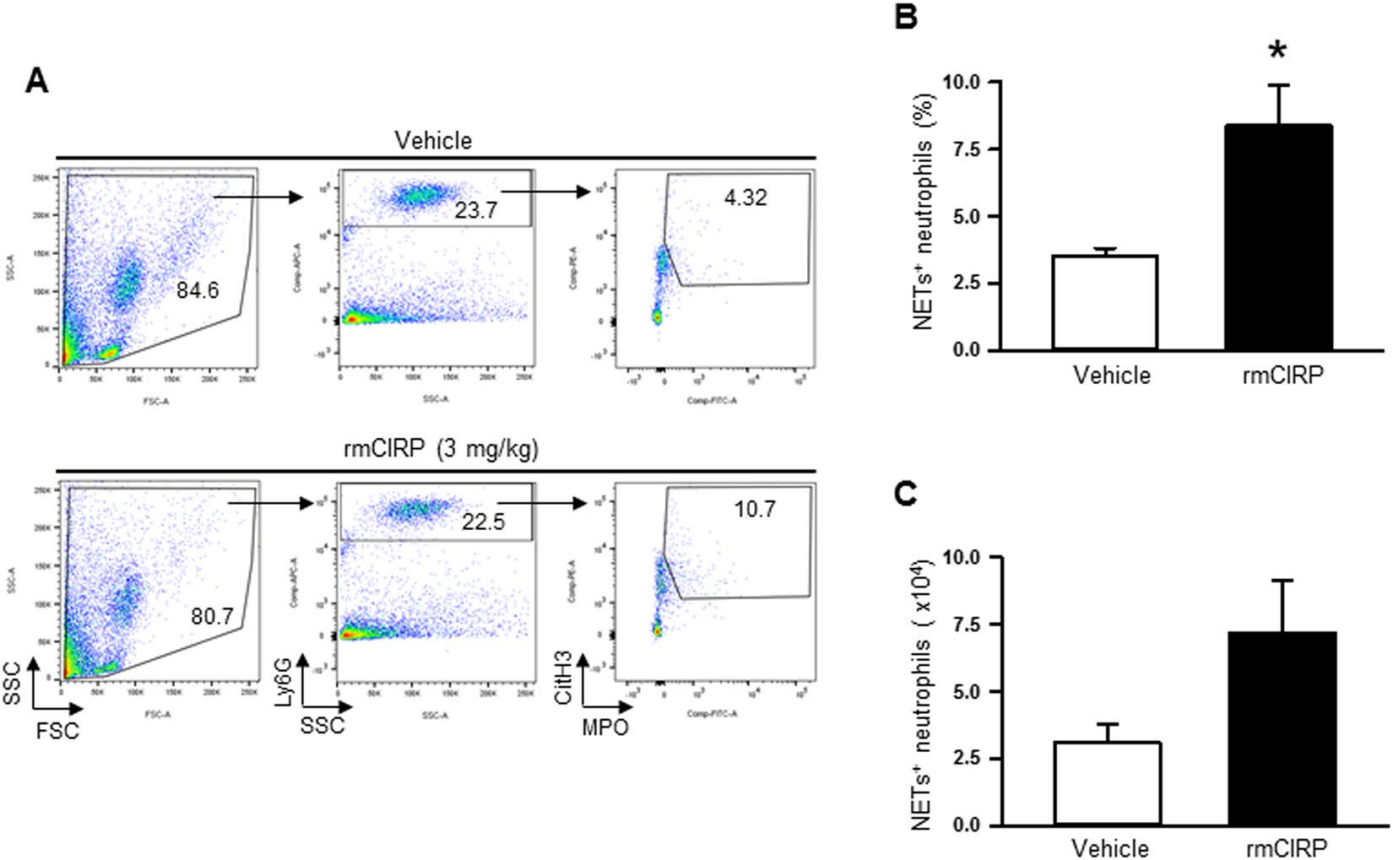
Assessment of NETs in the lungs of rmCIRP injected mice. After 5 h of rmCIRP or vehicle (normal saline) *i.t*. injection, lungs were perfused and harvested. NET formation in neutrophils isolated from lungs was quantified by flow cytometry by staining the cells with anti-mouse Ly6G, MPO, and CitH3 Abs. (**A**) Representative dot plots of the frequencies of NET forming neutrophils in lungs generated from two independent experiments are shown. Bar diagrams representing the quantitative mean values of the (**B**) frequencies and **(C)** numbers of NET forming neutrophils in the lungs are shown. Data are expressed as means ± SE (n = vehicle 4 and rmCIRP 6 mice) and compared using t-test (*p < 0.05 vs vehicle).

To determine whether CIRP can directly induce NETosis *in vivo*, rmCIRP was injected intratracheally into normal healthy WT mice. We injected 3 mg/kg BW of rmCIRP in mice, and this *in vivo* dose of rmCIRP was chosen from our previous studies^8,24^. We chose the strategy of delivering rmCIRP in mice through *i.t*. route, which was based on our previous study demonstrating *i.t*. delivered rmCIRP in mice to induce phenotypic changes of neutrophils^24^. Five hours after injection of rmCIRP, we found a significant increase in the frequencies and numbers of NET forming neutrophils in lungs by 58% and 57%, respectively, as compared to vehicle-injected mice **(**Fig. 2A–C**)**.

### *In vitro* treatment of BMDN with rmCIRP induces NETosis

**Figure 3:**
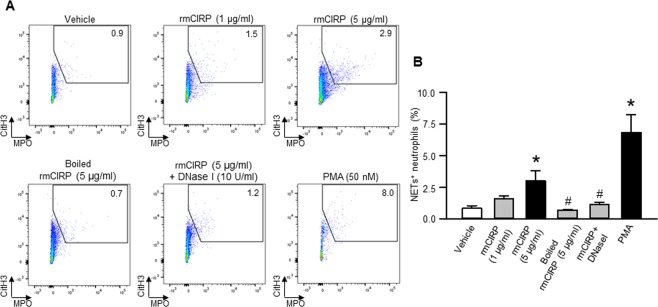
Assessment of NETosis in rmCIRP-treated neutrophils *in vitro*. A total of 1 × 10^6^ BMDN were treated with the indicated dose of rmCIRP, boiled rmCIRP (5 µg/ml of rmCIRP heated at 80 °C for 5 min), rmCIRP (5 µg/ml) + DNase I (10 U/ml; ThermoFisher Scientific) and phorbol 12-myristate 13-acetate (PMA, 50 nM, Sigma-Aldrich) for 4 h at 37 °C in 5% CO_2_. NETs in rmCIRP treated neutrophils were quantified by flow cytometry by staining the cells with anti-mouse Ly6G, MPO, and CitH3 Abs. (**A**) Representative dot plots of the frequencies of NET forming neutrophils generated from two independent experiments are shown. **(B)** Bar diagrams representing the quantitative mean values of the frequency of NET forming neutrophils are shown. Data are expressed as means ± SE (n = 4 samples in each group) and compared by using one-way ANOVA and SNK method (*p < 0.05 vs vehicle).

**Figure 4:**
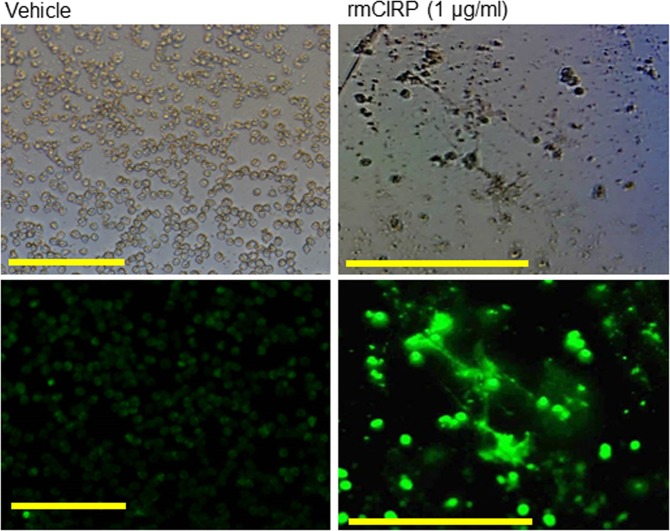
Assessment of NETs in rmCIRP-treated neutrophils by florescent microscope. A total of 1 × 10^5^ BMDN grown in culture slides were treated with rmCIRP (1 µg/ml) for 4 h at 37 °C in 5% CO_2_. The cells were fixed with 4% paraformaldehyde and then stained with SYTOX green (2 µM) for extracellular DNA detection, followed by the assessment using fluorescence microscope (bottom panels). Web-like structures were observed in rmCIRP treated neutrophils. Top panels show the images of their counterpart under light microscope. Scale bars indicate 100 µm; original magnification ×200.

To study whether CIRP can directly induce NETs *in vitro*, BMDN isolated from WT mice were treated with rmCIRP at various doses, denatured (boiled) rmCIRP, rmCIRP with DNase I, and PMA as positive control for 4 h, and then assessed NETosis. We found that treatment with rmCIRP significantly increased NET formation in the BMDN in a dose-dependent manner, in which the highest induction of NETosis by 2.95 fold was seen at 5 µg/ml of rmCIRP stimulation to BMDN compared to vehicle-treated control **(**Fig. 3A,B). BMDN treated with denatured rmCIRP had no effect on NET formation. Similarly, the DNase I treatment to BMDN significantly abolished the contents of rmCIRP-induced NETs, indicating the specificity of the NETs values formed by rmCIRP treatment **(**Fig. 3A,B). We also found that the BMDN treated with PMA significantly increased the frequencies of NET forming cells compared to PBS-treated BMDN **(**Fig. 3A,B). Since CIRP is an RNA-binding protein, we aimed to determine whether or not the extracellular RNA have any influence on CIRP-induced NET formation. BMDN were treated with either rmCIRP alone or together with exogenous mRNA isolated from mouse spleen in the presence or absence of RNase A simultaneously. We found that treatment of BMDN with rmCIRP significantly increased the frequencies of NET-forming neutrophils. However, when BMDN were treated with rmCIRP and exogenous mRNA together we found that the frequencies of NET-forming neutrophils were significantly lower than that of only rmCIRP-treated BMDN (Supplemental Fig. 3). Conversely, the simultaneous treatment of RNase A in rmCIRP and exogenous mRNA stimulated BMDN showed significant increase in the frequencies of NET-forming neutrophils compared to BMDN treated with rmCIRP and exogenous mRNA, suggesting that exogeneous mRNA might alter rmCIRP’s induction of NET-forming neutrophils (Supplemental Fig. 3). We further confirmed NETosis in BMDN following treatment with rmCIRP by their morphological changes characteristic of NETs under the microscopy. We observed web-like structures in rmCIRP-treated neutrophils, which were largely absent in the PBS-treated control **(**Fig. 4).

### Deficiency of CIRP leads to decreased expression of PAD4 in the lungs during sepsis

**Figure 5:**
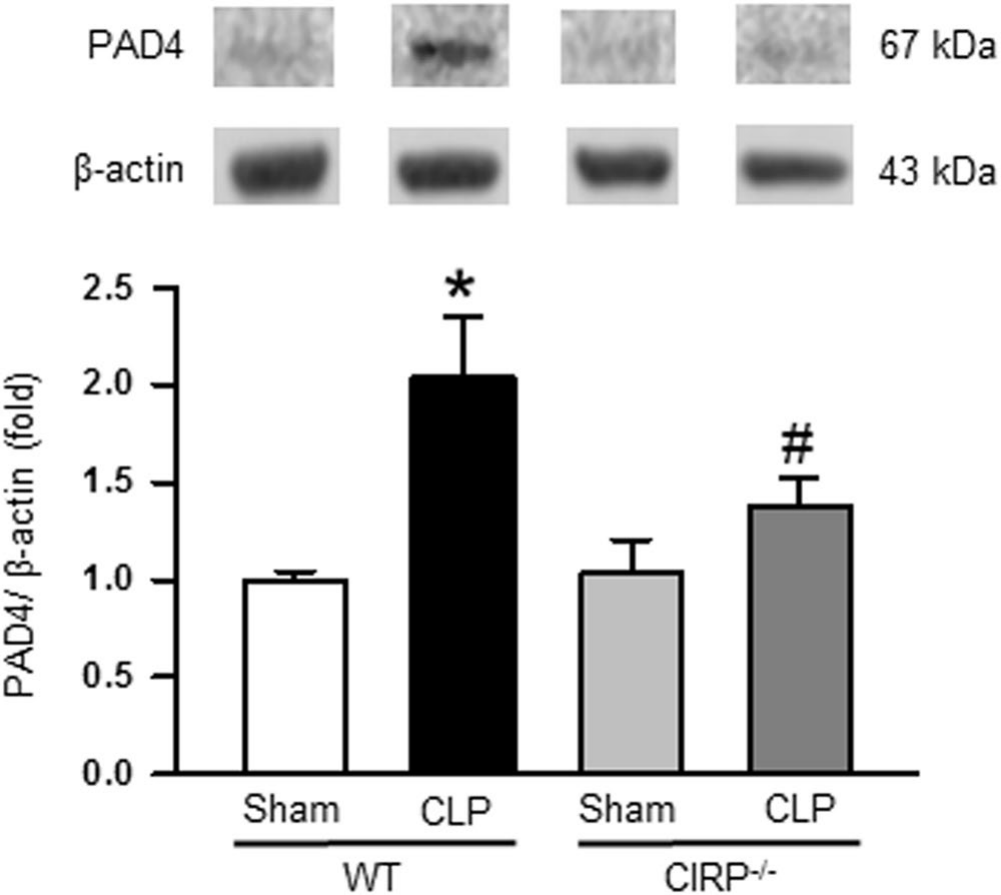
Assessment of PAD4 expression in the lungs during sepsis. Total protein was extracted from lung tissues and PAD4 expression at protein levels was measured by Western blotting. Each blot was quantified by densitometric analysis. PAD4 expression in each sample was normalized to β-actin expression and the mean values of WT sham groups were standardized as one for comparison. Representative blots for PAD4 and β-actin from three independent experiments are shown. The full-length gel from where the cropped bands are taken is displayed in Supplemental Fig. 4, in which the cropped bands are shown in yellow box. Data are expressed as means ± SE (n = WT sham 10, WT CLP 10, CIRP^−/−^ sham 10, and CIRP^−/−^ CLP 10 mice) and compared using one-way ANOVA and SNK method (*p < 0.05 vs WT sham, ^#^p < 0.05 vs WT CLP).

After 20 h of CLP, PAD4 expression in the lungs of WT mice was significantly increased by 1.9 fold as compared to the sham-operated mice, whereas the CIRP^−/−^ mice showed a significant decrease in the expression of lung PAD4 by 32% compared to WT CLP mice **(**Fig. 5, Supplemental Fig. 4).

### *In vivo* and *in vitro* treatment of rmCIRP induces PAD4 expression

**Figure 6:**
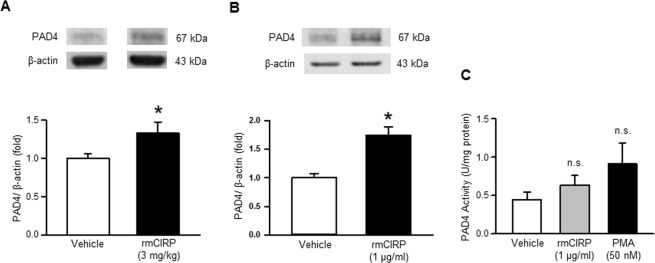
Assessment of PAD4 expression following rmCIRP stimulation *in vivo* and *in vitro*. (**A**) Total protein was extracted from the lung tissues of normal saline (vehicle) or rmCIRP-injected animals and PAD4 protein level was assessed by Western blotting. Each blot was quantified by densitometric analysis. PAD4 expression in each sample was normalized to β-actin expression and the mean values of vehicle treated groups were standardized as one for comparison. Representative blots for PAD4 and β-actin are shown. Data are expressed as means ± SE (n = vehicle 4, rmCIRP-treated 5 mice) and compared using Student’s *t*-test (*p < 0.05 vs vehicle). (**B**) A total of 2 × 10^6^ BMDN were treated with rmCIRP (1 µg/ml) for 4 h and then assessed the protein levels of PAD4 by Western blotting. PAD4 expression in each sample was normalized to β-actin expression and the mean values of vehicle treated groups was standardized as one for comparison. Representative blots for PAD4 and β-actin from three independent experiments are shown. The full-length gels from where the cropped bands are taken is displayed in Supplemental Figs 5 and 6, in which the cropped bands are shown in yellow box. Data are expressed as means ± SE (n = 10 samples in each group) and compared using Student’s *t*-test (*p < 0.05 vs vehicle). **(C)** PAD4 enzymatic assay. Total protein was extracted from a total of 1 × 10^6^ BMDN treated with PBS, rmCIRP (1 µg/ml) or PMA (50 nM) and analyzed the activity of the PAD4 enzyme as described in the Materials and Methods. Data are expressed as means ± SE (n = 5 samples in each group) and compared using one-way ANOVA and SNK method (n.s. = non-significant).

We assessed the expression of PAD4 in the lungs following treatment of WT mice with either vehicle or rmCIRP. PAD4 expression in the lungs of rmCIRP injected-mice was significantly increased by 1.7 fold, compared to vehicle-injected mice **(**Fig. 6A, Supplemental Fig. 5). We also assessed PAD4 expression in BMDN after treatment with rmCIRP *in vitro*. We found that rmCIRP treatment in BMDN significantly increased the protein levels of PAD4 by 1.3 fold compared to vehicle-treated control **(**Fig. 6B, Supplemental Fig. 6). We performed a pilot study to show the status of PAD4 enzyme activity in PBS- or rmCIRP-treated BMDN (Fig. 6C). We found that treatment of BMDN with rmCIRP at 1 µg/ml increased the enzymatic activity of PAD4 by 42% compared to PBS (vehicle) treated group (Fig. 6C). As a positive control, PMA treatment to BMDN also increased the PAD4 enzyme activity in BMDN (Fig. 6C).

## Discussion

CIRP is a novel DAMP causing exaggerated inflammation and injury to the lungs in murine sepsis as well as after direct administration of rmCIRP into the mice^4,10^. We previously showed that CIRP induces neutrophil infiltration, endothelial cell dysfunction by upregulating NLRP3 inflammasome and endoplasmic reticulum (ER) stress in lungs to cause ALI^8–10,25^. In the present study, we demonstrated that CIRP induced NET formation in the lungs during sepsis and following treatment of healthy mice with rmCIRP. We further revealed that CIRP’s induction of murine primary BMDN directly induced NETosis. PAD4 has been shown to play a pivotal role in NET formation^13^. Our present study demonstrated that CIRP deficiency led to decreased PAD4 expression and subsequent NET formation in the lungs during sepsis. We also determined CIRP’s induction of the PAD4 expression in BMDN exposed *in vitro* to rmCIRP and in the lungs of healthy mice injected with rmCIRP. Collectively, these data clearly suggest that CIRP functions as a novel inducer of NETs by upregulating PAD4 expression in sepsis.

Intratracheal instillations deliver solutes directly into the lungs. This procedure targets the delivery of the instillate into the distal regions of the lung, and is therefore often utilized in studies aimed at studying alveoli^26,27^. In this study, we injected mice with rmCIRP directly into the lungs *i.t*., which targeted the lung tissues for exerting pro-inflammatory effects. Our previous studies showed that direct injection of rmCIRP *i.t*. or *i.v*. upregulated the expression of pro-inflammatory cytokines, chemokines and increased the infiltration of neutrophils in lungs to cause ALI in mice^8,24^.

Here, we demonstrated elevated NET formation in the lungs during polymicrobial sepsis by flow cytometry, using two key components of NETs, citrullinated histone H3 and MPO. Our approach of determining NETs by flow cytometry is consistent with a recent study using CLP-induced septic mice to determine circulating cell free DNA (cf-DNA) and NETs in bronchoalveolar lavage fluid (BALF) by staining extracellular DNA with SYTOX Green^28^. The gold standard tool for NETs quantification has not been well established yet. Since the major components of NETs are extracellular DNA, histones (H2A, H2B, H3, H4, and H1) and several granule proteins such as neutrophil elastase (NE) or MPO^11^, presence of these components outside neutrophils serve as potential markers for the detection and quantification of NETs. As these molecules are mainly present inside the neutrophils, while detecting NETs by flow cytometry or microscopy these molecules were stained outside the cells without permeabilization of the cells. cf-DNA has been frequently used for the quantification of NETs in biological fluids including cell culture supernatants *in vitro* and sera or tissue fluids *in vivo*. However, further consideration about the source of the extracellular DNA and their stability outside the cells is required^29^.

Due to its high sensitivity, the flow cytometry based detection and quantification approach seems to be a promising tool for NETs assay^20,28,29^. In this study, we quantified NET formation by flow cytometry following a recent protocol^20^. The flow based NETs detection method was validated by microscopic assay in murine and human blood samples^20^. NETs are fragile, therefore some efforts are required in order to detect and quantify them efficiently^30^. In our study, we used neither DNase during lung cell isolation, nor did we use fetal bovine serum (FBS) containing medium to avoid possible NETs disruption.

We demonstrated elevated levels of PAD4 in the lungs following sepsis. There are five mammalian PAD family members (PADs 1-4 and 6) that have been described, which show tissue- and cell-specific distribution^31^. PAD4 is expressed in granulocytes and catalyzes citrullination of histones leading to chromatin decondensation, which is thought to be essential process for NETs release^13^. PAD4^−/−^ neutrophils cannot form NETs, and pharmacological inhibition of PAD4 prevents NET formation in mice^15,16^. Thus, we considered that measuring PAD4 expression in neutrophils and lung homogenates would keep validate NET formation indirectly and that using CitH3 as one of the surface markers of NETs in flow cytometric analysis kept our strategy consistent during this study. We determined CIRP to be a novel inducer of NETs by increasing the expression of PAD4 in the neutrophils. Using a genetic approach for targeting CIRP, we found that the CIRP^−/−^ mice produced significantly decreased levels of NETs in the lungs compared to WT mice in sepsis by decreasing the expression of PAD4. Our previous studies showed that the pharmacologic approaches targeting CIRP, neutralizing Ab against CIRP or CIRP-derived small peptide (C23) for example, attenuate local and systemic inflammation and remote organ inflammation and injuries in sepsis^4^. These premises strongly suggest that anti-CIRP Ab or C23 treatment might abrogate NET formation by inhibiting PAD4 expression in sepsis. Besides its role in citrulination of proteins and NET formation, PAD4 has been shown to regulate the proliferation of multipotent progenitors in the bone marrow^32^. In our study, we primarily focused on the function of PAD4 in CIRP-induced NETosis. However, further elucidation of the role of CIRP-induced PAD4 on hematopoiesis, progenitor cell proliferation and differentiation will increase our understandings of the impact of PAD4’s expression on cell physiology and homeostasis during inflammation. It should be noted that these findings are based on the results of experiments using BMDN and lung neutrophils isolated from male 8-12 week old mice. The behavior of neutrophils toward NET formation possibly depend on their species and ages^33,34^. It has been reported that murine neutrophils make NETs more slowly and less efficiently than human cells^35^, and PAD4 may be important for murine NET release but not for human^35,36^.

To date, besides pathogens (bacteria, fungi, parasites, viruses) and their related PAMPs (LPS), several endogenous stimuli, such as DAMPs like high mobility group box 1 protein (HMGB1), or chemokines and cytokines (e.g. IL-8, TNF-α, IL-1β) have been reported to serve as the inducers of NETosis^11,37,38^. At least two different forms of NETosis termed “suicidal” and “vital” has been reported^39,40^, and several process are estimated to be implicated in these NET formation, such as reactive oxygen species (ROS) production, PAD4 dependent hypercitrulination of histone H3 followed by chromatin decondensation, or NE and MPO released from granules into cytosol^41^. Thus, it is currently considered that diverse stimuli induce NETs through several processes^35,36,42^. Although we detected MPO and CitH3 as NETs surface markers and demonstrated PAD4 up-regulation in rmCIRP treated neutrophils, suggesting a potential link between PAD4 mediated histone citrullination and NETosis in CIRP-treated condition, the mechanism by which CIRP promotes which form of the NETosis was not elucidated in this study. It has been shown that NADPH oxidase activation and ROS generation can induce PAD4 activation^13^.

In the present study, we did not determine the NAD(P)H oxidase activity and the type of NETosis after stimulation of BMDN with rmCIRP, which we confess to be the limitations of our study. NAD(P)H oxidase is a complex of multimeric enzymes consisting of 5 subunits, gp91*phox*, p22*phox*, p47*phox*, p67*phox*, and small GTPase Rac^43^. In our previous study, we found that stimulation of lung vascular endothelial cells with rmCIRP increased the binding of gp91*phox* to phosphorylated p47*phox*, indicating NAD(P)H’s induction^10^. We therefore speculate that treatment of neutrophils with rmCIRP may induce NAD(P)H activation and subsequently the ROS production. In the suicidal NETosis, neutrophils activate the NAD(P)H oxidase complex through protein kinase C (PKC)/Raf/MERK/ERK, lead to the activation of PAD4^40,44^. Whereas, in the vital NETosis, neutrophils release NETs without exhibiting a loss of nuclear or plasma membrane and it occurs independently of ROS. Since CIRP was previously shown to induce NAD(P)H oxidase and subsequently ROS production in the endothelial cells^10^, it is presumable that CIRP might induce suicidal NETosis. Future studies revealing how CIRP induces PAD4 expression and forms suicidal or vital form of NETosis will establish a new paradigm on CIRP’s role in neutrophil biology.

In our study, we found a 2.7 fold induction of NETosis in rmCIRP-treated BMDN. It has been shown that only a small fraction (20-30%) of neutrophils in a culture make NETs, which is dependent on the nature of stimuli^45^. It has not yet been understood whether a specialized subset of neutrophils can induce NETosis upon stimulation. We recently demonstrated that CIRP-induced ICAM-1^+^ phenotype of neutrophils can promote elevated NET formation in the lungs in murine sepsis, which might suggest a link between phenotypic variation and increased NET formation during sepsis^8^. In addition, relatively small induction of NETosis in *ex vivo* cell cultured condition compared to *in vivo* condition may suggest the substantial role of crosstalk between neutrophils and other cell types such as endothelial cells, platelets or other immune cells in the NET formation under *in vivo* condition^46,47^.

It has been reported that PMA-induced NETs directly promote epithelial and endothelial cell death *in vitro*^47^, and LPS-induced NETs directly activate macrophage to release IL-1β^48^, however, the direct effect of CIRP-induced NETs on lung epithelial, endothelial and various innate and adaptive immune cells was not examined in this study. NETs are considered to be a double-edged sword in sepsis^49^. NETs might be beneficial to eliminate pathogens, especially pathogens which are too large to be phagocytosed via immobilizing and possibly killing them^50,51^. In contrast, NETs are deleterious to the host via promoting inflammation, hypercoagulability and tissue injury in sepsis^14,28,52^, as well as in sterile inflammation and its mediated several autoimmune diseases in which NETs are estimated to play a substantial role for their pathophysiology^53^. Increasing evidence has demonstrated the detrimental association between NETs and ALI evidenced by NETs inhibition in several animal models, such as sepsis^14,23^, LPS injection^14,54^, and in ventilator-induced lung injury^55^, which may suggest an expanding potential of NETs-targeted therapy with or without administration of antibiotics^14,15^. Moreover, these previous studies further suggest that our new findings indicate a possibility of CIRP to develop ALI through NET formation in sepsis.

CIRP has been shown to be elevated in the plasma of human and murine sepsis^4,5^, also in the lungs of septic mice^8^. Deficiency or pharmacological inhibition of CIRP attenuates systemic inflammation and organ injury, thus improving overall survival in the animal sepsis model^4,8,56^. Future studies on targeting CIRP by its neutralizing Abs or small peptide to attenuate NET formation in the lungs during sepsis or in sterile inflammation will introduce novel therapeutic approaches in these deadly infectious diseases.

In summary, we reported a novel function of CIRP to generate NETs in the lungs during sepsis evidenced by flow cytometric analysis identifying CitH3 and MPO double positive neutrophils as NETs and by showing PAD4 upregulation in neutrophils or lung homogenates. Our findings provide new evidence which demonstrates a novel pathophysiological role of CIRP on promoting ALI during sepsis.

## Supplementary information

Dataset 1
