# Supplementary material for: Cold-inducible RNA-binding Protein Induces Neutrophil Extracellular Traps in the Lungs during Sepsis
Source: Sci Rep. 2019 Apr 18;9:6252. doi: 10.1038/s41598-019-42762-1 (PMC6472421; doi:10.1038/s41598-019-42762-1)

Cold-inducible RNA-binding Protein Induces Neutrophil Extracellular Traps in the Lungs during Sepsis

Yasumasa Ode[1], Monowar Aziz[1], Hui Jin[1], Adnan Arif[1], Jonathan G. Nicastro[1], Ping Wang[1,2*]

[1]Center for Immunology and Inflammation, The Feinstein Institute for Medical Research, Manhasset, NY; [2]Department of Surgery and Molecular Medicine, Donald and Barbara Zucker School of Medicine at Hofstra/Northwell, Manhasset, NY.

Supplemental Fig 1

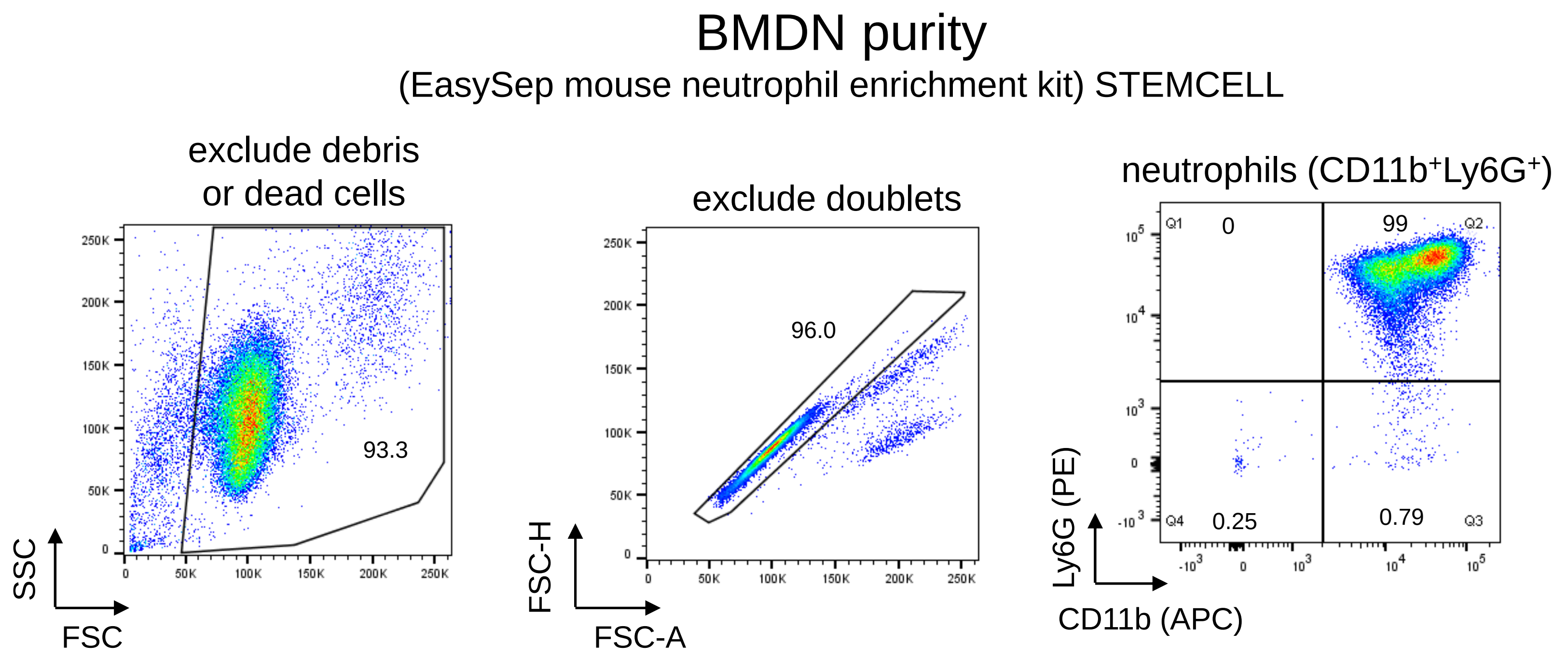

Supplemental Fig 2

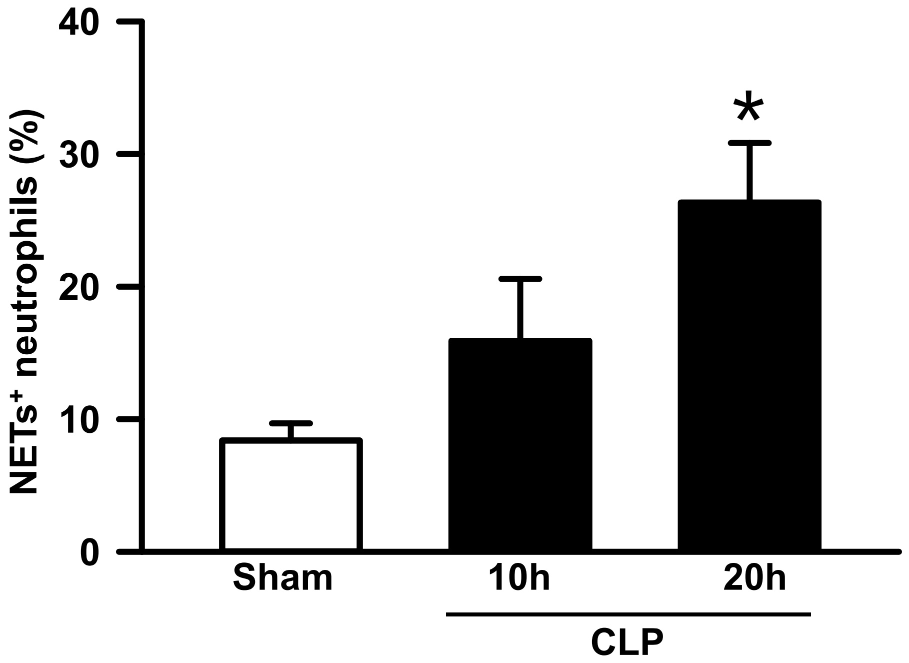

At 10 and 20 h of CLP or sham operation, lungs were perfused and harvested from WT mice. NET forming neutrophils in the lungs was detected by flow cytometry by staining the single cell suspension with anti-mouse Ly6G, MPO, and CitH3 Abs. Bar diagram representing the quantitative mean values of the frequencies of NET forming neutrophils in the lungs generated from three independent experiments are shown. Data are expressed as means ± SE and compared using one-way ANOVA and SNK method (*$p<0.05$ vs sham). N=4-5 mice/group (Sham=4; 10 h CLP=4; 20 h CLP=5 mice).

Supplemental Fig 3

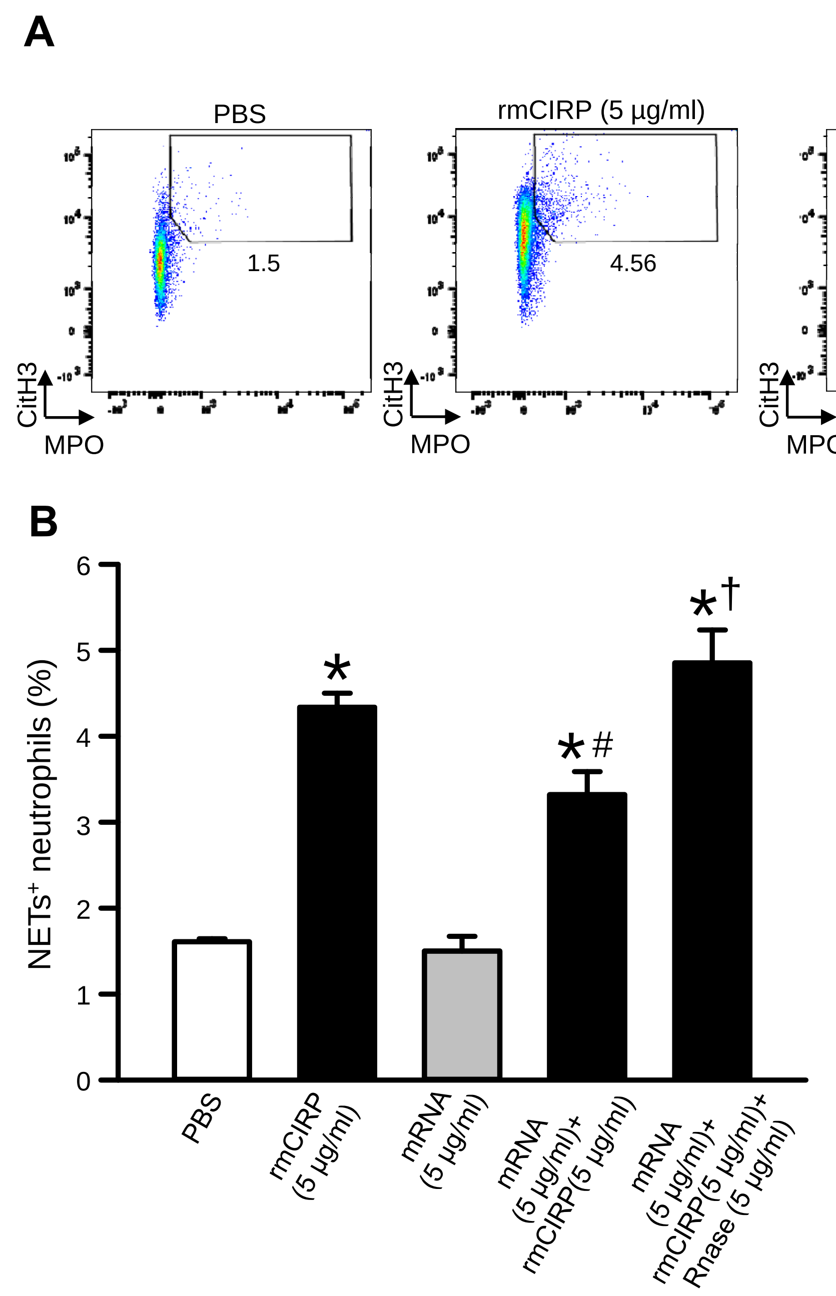

A total of 1 × 10^6 purified BMDN were treated with PBS (vehicle), rmCIRP (5 µg/ml), exogenous mRNA (5 µg/ml), exogenous mRNA (5 µg/ml) + rmCIRP (5 µg/ml) and exogenous mRNA (5 µg/ml) + rmCIRP (5 µg/ml) + Rnase A (5 µg/ml; Thermo Fisher Scientific, Waltham, MA). After 4 h the cells were fixed with 2% paraformaldehyde and stained with anti-citH3 and anti-MPO Abs and subjected to flow cytometric assay. Exogenous mRNA was isolated from mouse spleen using Rneasy Mini Kit (Qiagen, Hilden, Germany). **(A)** Representative dot plots and **(B)** quantitative bar diagram are shown. Data are expressed as means ± SE and compared using one-way ANOVA and SNK method (*p<0.05 vs PBS, $^{\#}$p<0.05 vs rmCIRP, †p<0.05 vs mRNA+rmCIRP). N=5 samples/group.

Supplemental Fig 4

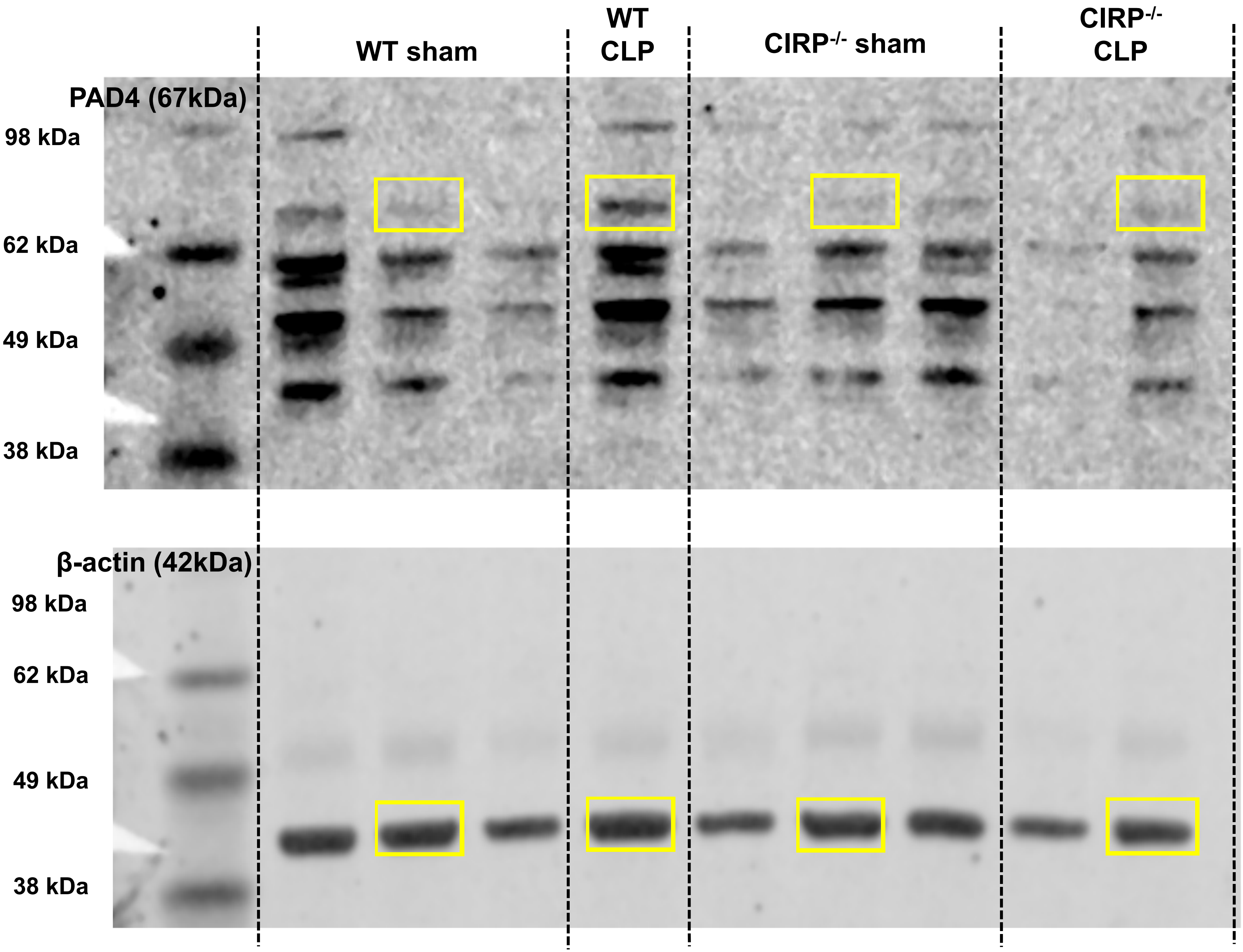

Supplemental Fig 5

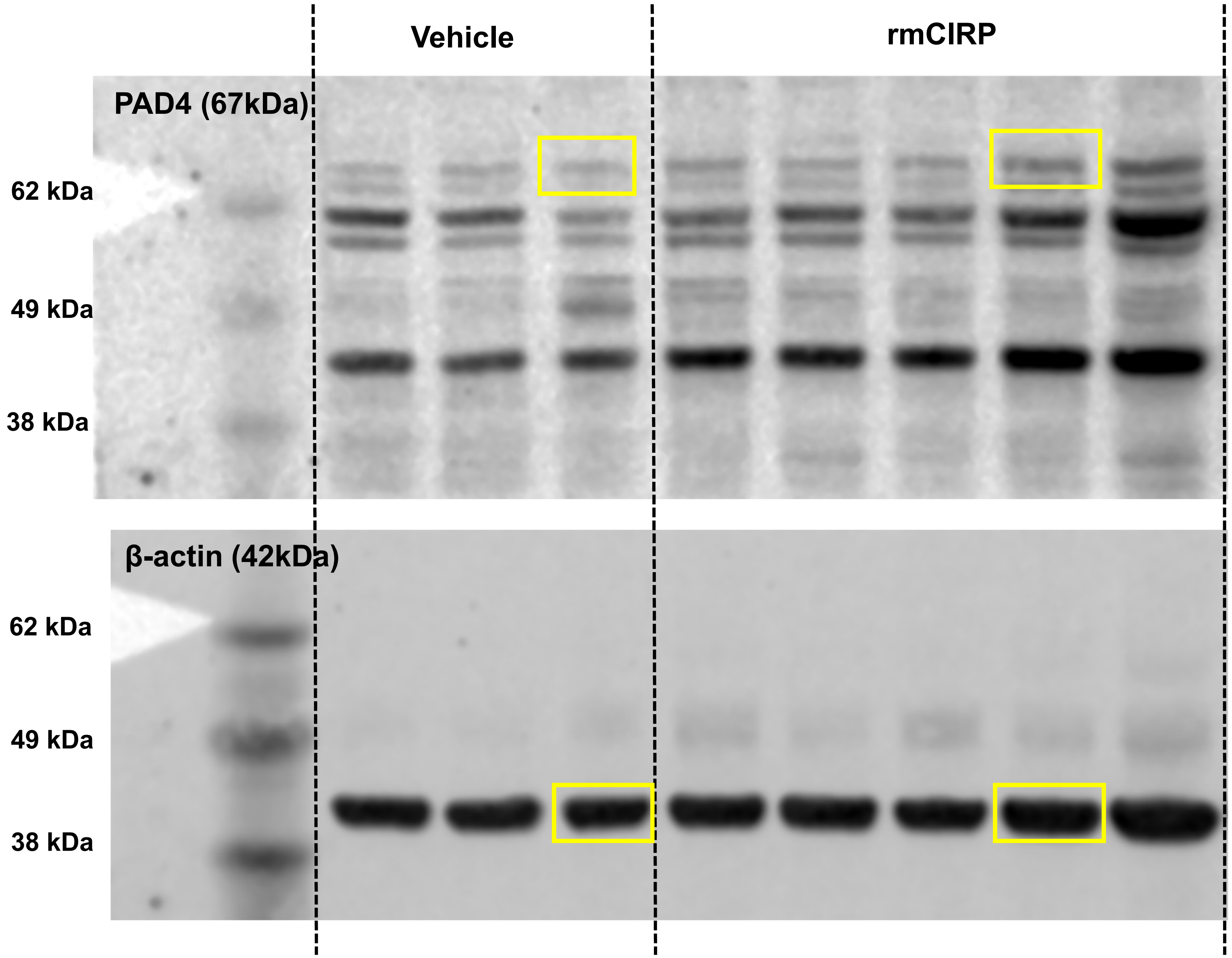

Supplemental Fig 6

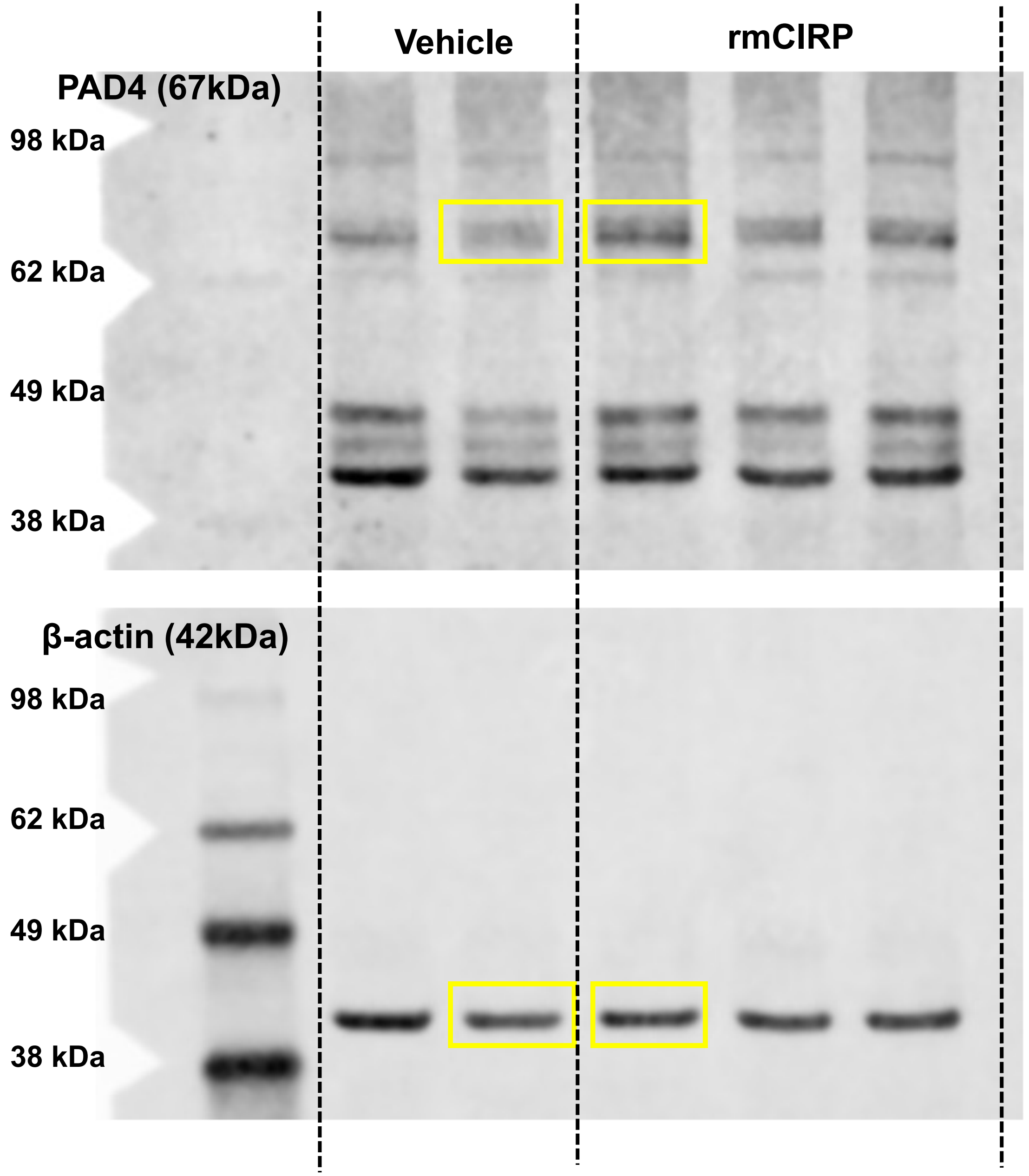

Supplement: Supplementary file 1 — Dataset 1 [file 41598_2019_42762_MOESM1_ESM.pdf]
